# Molecular pathology in diagnosis and prognostication of head and neck tumors

**DOI:** 10.1007/s00428-023-03731-2

**Published:** 2024-01-13

**Authors:** Alena Skálová, Martina Bradová, Michael Michal, Elaheh Mosaieby, Natálie Klubíčková, Tomáš Vaněček, Ilmo Leivo

**Affiliations:** 1 Sikl’s Department of Pathology, Faculty of Medicine in Pilsen, Charles University, E. Benese 13, 305 99 Pilsen, Czech Republic; 2 Bioptic Laboratory, Ltd, Pilsen, Czech Republic; 3 Molecular and Genetic Laboratory, Bioptic Laboratory, Ltd, Pilsen, Czech Republic; 4 Institute of Biomedicine, Pathology, University of Turku and Department of Pathology, Turku University Hospital, Turku, Finland

**Keywords:** Salivary gland, Sinonasal tumor, Soft tissue, Head and neck, Molecular diagnostics, Next-generation sequencing

## Abstract

Classification of head and neck tumors has evolved in recent decades including a widespread application of molecular testing in tumors of the salivary glands, sinonasal tract, oropharynx, nasopharynx, and soft tissue. Availability of new molecular techniques allowed for the definition of multiple novel tumor types unique to head and neck sites. Moreover, the expanding spectrum of immunohistochemical markers facilitates a rapid identification of diagnostic molecular abnormalities. As such, it is currently possible for head and neck pathologists to benefit from a molecularly defined classifications, while making diagnoses that are still based largely on histopathology and immunohistochemistry. This review highlights some principal molecular alterations in head and neck neoplasms presently available to assist pathologists in the practice of diagnosis, prognostication and prediction of response to treatment.

## Introduction

Classification of head and neck tumors has expanded dramatically in recent decades with identification of many new entities and widespread application of molecular testing in tumors of the salivary glands, sinonasal tract, oropharynx, nasopharynx, and soft tissue of the head and neck. Molecular testing has disclosed pathogenetic processes of well-established but previously enigmatic entities, and clarified the relationships between various neoplasms. The current 5th edition of the WHO Classification of Head and Neck Tumours relies heavily on molecular data to support the inclusion of several new tumor entities and their subtypes, and to provide prognostic and pathogenetic information [1]. Furthermore, the description of molecular alterations in various subgroups of tumors and the application of molecular testing in large case cohorts in a research setting has substantially clarified the boundaries of many existing histologic entities and allowed for the identification of specific morphologic and immunohistochemical features of new tumor types. On the other hand, it must be emphasized that this molecular revolution in head and neck pathology has not made pathologists entirely dependent on molecular testing in making their diagnoses. An expanding spectrum of immunohistochemical markers based on new molecular information has improved diagnostic options using immunohistochemistry, and such markers also facilitate a rapid identification of useful diagnostic molecular tests. Thus, head and neck pathologists currently benefit from a molecularly defined classification, while making diagnoses that are still based largely on histopathology and immunohistochemistry.

In recent years, considerable progress in salivary gland and sinonasal tumor taxonomy has taken place with the discovery of tumor type-specific fusion oncogenes generated by chromosomal translocations. This review covers molecular alterations important in head and neck neoplasms from a practical viewpoint in diagnosis, prognostication and prediction of response to treatment.

## Salivary gland neoplasms

Salivary gland tumors represent a challenging field in diagnostic head and neck pathology. These rare tumors exhibit a diverse array of morphological features, comprising almost 40 subtypes in the current World Health Organization (WHO) Classification of Head and Neck Tumours [1]. Traditionally, salivary neoplasms are diagnosed on morphological grounds, with the help of ancillary stains and immunohistochemistry. Several salivary gland neoplasms are characterized by recurrent genomic alterations, particularly gene fusions. These include fusions of the *ETV6* gene in secretory carcinoma (SC) [2–4], *MYB/MYBL1*::*NFIB* in adenoid cystic carcinoma (AdCC) [5], *CRTC1/CRTC3*::*MAML2* in mucoepidermoid carcinoma (MEC) [6, 7], and *EWSR1::ATF1* in hyalinizing clear cell carcinoma [8] as the most frequent and best characterized gene fusions (Table 1).

**Table 1:** Immunohistochemical characteristics and genetic alterations and in salivary gland tumors reviewed in this article

Tumor type	IHC	Molecular genetic	Chromosomal region
*Benign epithelial tumors*			
Pleomorphic adenoma	PLAG1, HMGA2	*PLAG1* rearrangements	8q12
	SOX10, p63	HMGA2 rearrangements	12q13-15
*Malignant epithelial tumors*			
Mucoepidermoid carcinoma	p63, p40, mucicarmine, AMP	*CRTC1::MAML2*	t(11;19)(q21;p13)
		*CRTC3::MAML2*	t(11;15)(q21;q26)
		*EWSR1::POU5F1*	t(6;22)(p21;q12)
Adenoid cystic carcinoma	Biphasic, MYB, CD117	*MYB::NFIB*	6q22-23
		*MYBL1::NFIB*	8q13
		*NOTCH* mutations	9q34.3
Secretory carcinoma	S100, SOX10, mammaglobin, GAT3	*ETV6::NTRK3*	t(12;15) (p13;q25)
		*ETV6::RET*	t(12;10) (p13;q11)
		*ETV6:MET*	t(12;7) (p13;q31)
		*ETV6::MAML3*	t(12;4) (p13;q31)
		*VIM::RET*	t(10;10) (p13;q11)
		*CTNNA1*::*ALK*	t(11;20)
		*PRSS1* mutations	7q35
Microsecretory adenocarcinoma	S100, SOX10, p63	*MEF2C::SS18*	t(5q14.3) (18q11.2)
Polymorphous adenocarcinoma			
* Classic subtype*	S100, SOX10, CK7, p63 + /p40-	*PRKD1 mutations*	14q12
* Cribriform subtype*	S100, SOX10, CK7, p63-/p40-	*PRKD1* fusion	14q12
		*PRKD2* fusion	19q13.2
		*PRKD3* fusion	2q22.2
Hyalinizing clear cell carcinoma	CK7, CK19, CK14, CAM5.2, EMA, p63, p40, CK5/6	*EWSR1::ATF1*	t(12;22) (q21;q12) t(10;22)(p11;q12)
		*EWSR1::CREM*	
Salivary duct carcinoma	AR, CK7, HER2, GATA3		17q21.1
		*HER2* amplification	8p11.23
		*FGFR1* amplification	17p13.1
		*TP53* mutations	3q26.32
		*PIK3CA* mutations	11p15.5
		*HRAS* mutations	Xq12
		*AR* copy gain	10q23.31
		*PTEN* loss	9p21.3
		*CDKN2A* loss	8q12
		*PLAG1* rearrangements*	12q13-15
		*HMGA2* rearrangements*	
Epithelial-myoepithelial carcinoma	Biphasic; RAS Q61R	*HRAS* mutations (codon 61)	11p15.5

*- in case of preexisting pleomorphic adenoma

Excluded entities without diagnostic molecular genetic characteristics

Adapted from:

WHO Classification of Tumours Editorial Board. Head and neck tumours. Lyon (France): International Agency for Research on Cancer; forthcoming. (WHO classification of tumours series, 5th ed.; vol. 9). https://publications.iarc.fr

Skalova A, Hyrcza MD, Leivo I. Update from the 5th Edition of the World Health Organization Classification of Head and Neck Tumors: Salivary Glands. *Head Neck Pathol.* 2022;16:40–53

## Molecular alterations with prognostic and predictive significance

**Fig. 1:**
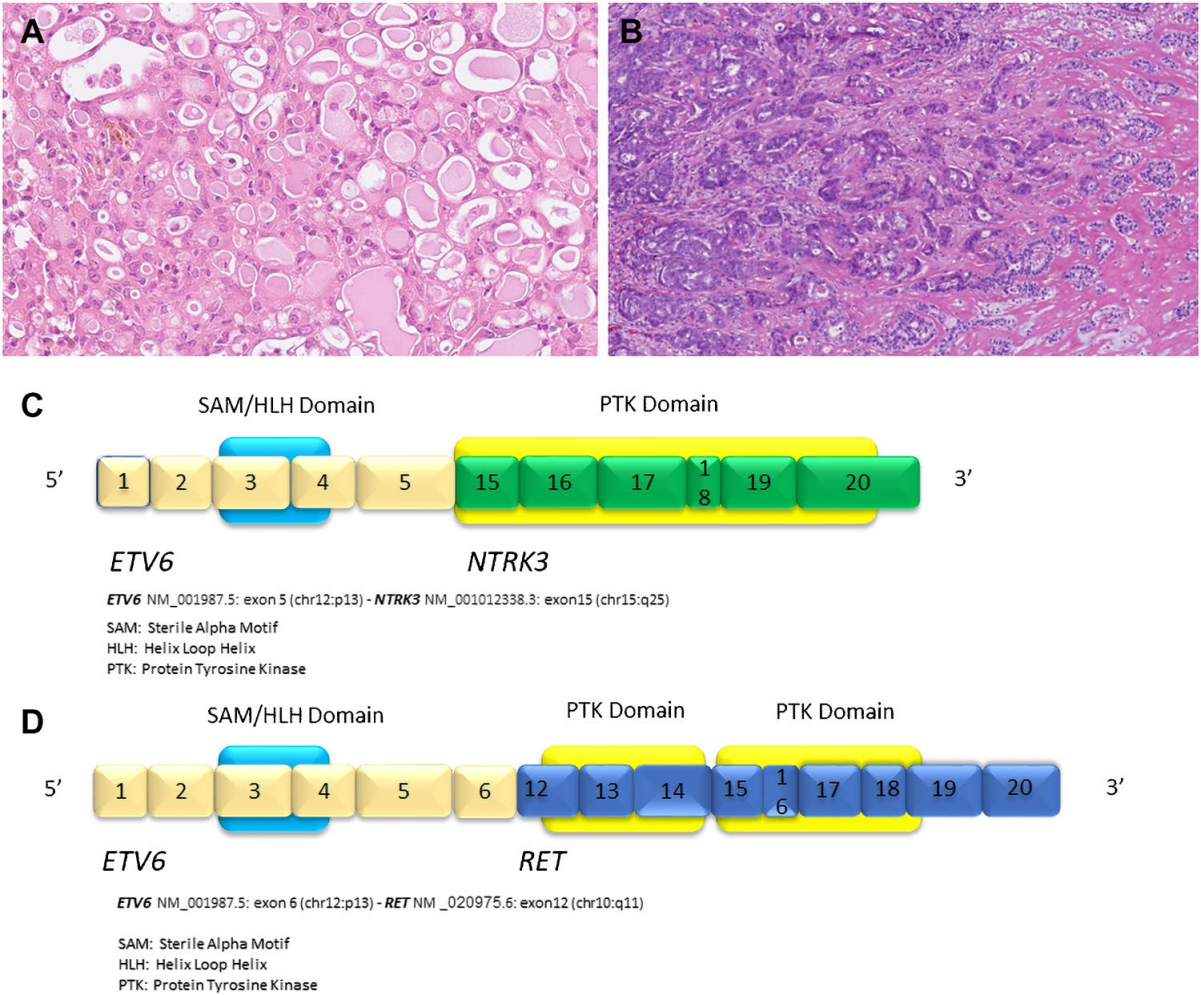
Secretory carcinoma (SC) of grade 1 with classical microcystic morphology with production of eosinophilic material **(A)**. SC of grade 3 morphology forming solid nests with minimal or no secretion **(B).** The fusions joining of *ETV6* gene exon 5 with *NTRK3* gene exon 15 **(C)** and *ETV6* gene exon 6 with *RET* gene exon 12 **(D)** are illustrated. Protein domains are depicted

**Salivary gland secretory carcinoma** (SC), previously called mammary analogue SC, is a mostly low-grade malignancy characterized by well-defined morphology and an immunohistochemical (Fig. 1A-D) and genetic profile identical to SC of the breast [2]. Translocation t(12;15)(p13;q25) resulting in the *ETV6::NTRK3* gene fusion is characteristic for SC, along with S100 protein and mammaglobin immunopositivity. The spectrum of genetic alterations in rare cases of SC continues to evolve including cases with *ETV6::RET* [3], *ETV6::MET* [9], and *VIM::RET* [10] fusions. Other rare alterations in low-grade SC include *CTNNB1::ALK* fusion [11] and dual fusions of *ETV6::NTRK3* and *ETV6:: MAML3* in the same tumor [12]. All these rearrangements can be used as diagnostically useful molecular markers in establishing the diagnosis of SC. Immunohistochemical positivity for S100, SOX10 and mammaglobin is a practical way to diagnose SC, and molecular testing may not be needed in most cases.

Furthermore, in cases with *ETV6::NTRK3* and *ETV6::RET* gene fusions in SC the introduction of targeted therapies using NTRK [13–15] and RET inhibitors [16] has implicated these fusions also as relevant predictive markers.

**Fig. 2:**
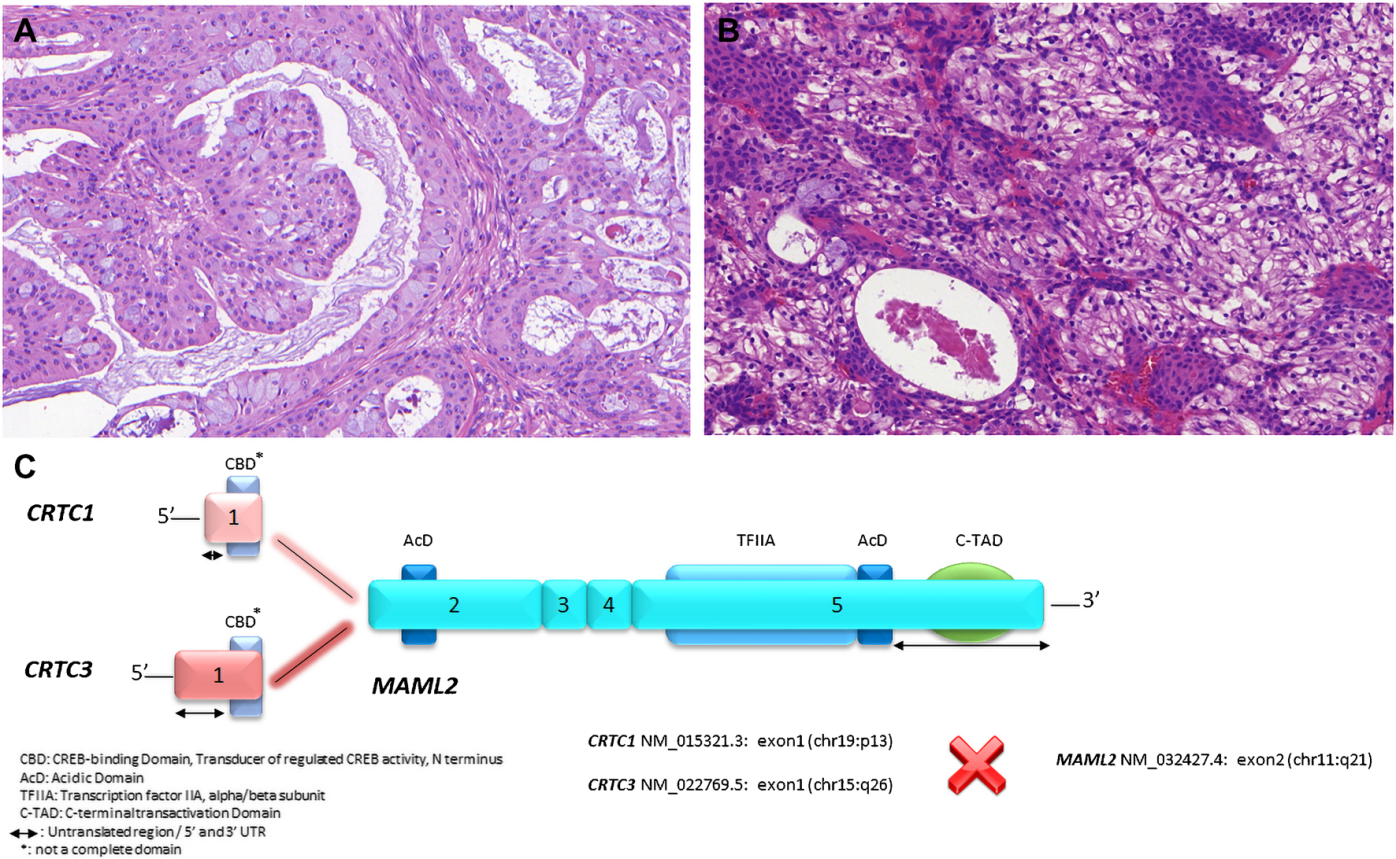
Classical mucoepidermoid carcinoma (MEC) consisting of cystic structures with plenty of mucoid cells settled within intermediate cells **(A)**. Clear cell variant of MEC with solid morphology and only few mucoid cells **(B)**. The fusions joining of *CRTC1* or *CRTC3* genes both exon 1 with *MAML2* gene exon 2 **(C)** are illustrated. Protein domains are depicted

**Salivary mucoepidermoid carcinoma** (MEC) is a malignant salivary gland neoplasm characterized by mucous, intermediate and epidermoid (squamoid) tumor cells forming cystic and solid growth patterns, and usually associated with *MAML2* gene rearrangement. Most MECs harbor a tumor type-specific translocation at t(11;19)(q21;p13) resulting in *CRTC1::MAML2* fusion gene [6, 7, 17]. Rare cases display a t(11;15)(q21;q26) translocation with *CRTC3::MAML2* fusion [18] (Fig. 2A-C) or a highly unusual t(6;22)(p21;q12) translocation with *EWSR1::POU5F1* fusion [19]. The *CRTC1::MAML2* fusion is seen in most low- and intermediate-grade and some high-grade tumors. *CRTC1/3::MAML2* fusions were originally considered as a biomarker for favorable overall survival in patients with salivary gland MEC [7]. More recent studies, however, have challenged these conclusions and current grading systems have not been fully satisfying in predicting the aggressiveness of MECs. A positive FISH-result for *MAML2*-rearrangement can be highly useful in confirming the diagnosis of MEC in difficult cases. Further evidence may be gained from NGS and PCR testing. However, negative FISH results are inconclusive, and cannot be used in differential diagnosis. The immunohistochemical profile of MEC includes positivity for p63, p40, and may be useful in differential diagnosis from other salivary gland tumors. In one recent study, *KMT2A* gene rearrangements in MECs identified aggressive tumor behavior regardless of histologic grade obtained with conventional grading systems [20]. Furthermore, these results may suggest the possibility of treating *KMT2A*-rearranged MECs using azacytidine and venetoclax, as these treatments have shown promising outcomes in the treatment of *KMT2A*-rearranged leukemias [21].

**Fig. 3:**
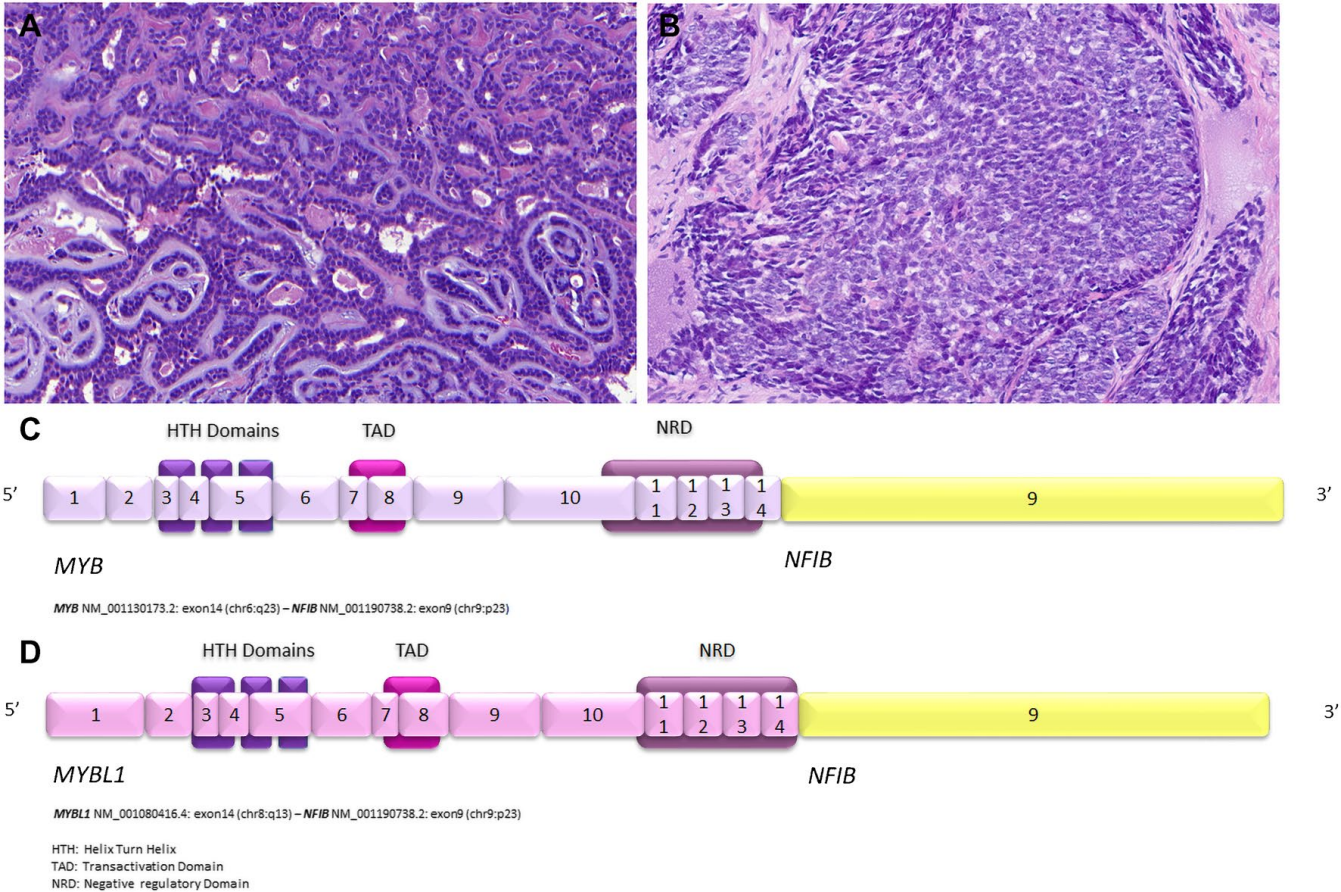
Adenoid cystic carcinoma with cribriform to tubular morphology containing basophilic matrix and reduplicated basement membrane material **(A)**. Solid pattern characterized by dense cellular tumour nests without pseudocystic spaces, this case showed *NOTCH1* gene mutation **(B)**. The fusions joining of *MYB* gene exon 14 with *NFIB* gene exon 9 **(C)** and *MYBL1* gene exon 14 with *NFIB* gene exon 9 **(D)** are illustrated. Protein domains are depicted

**Adenoid cystic carcinoma** (AdCC) is an invasive carcinoma composed of epithelial and myoepithelial neoplastic cells arranged in tubular, cribriform, and solid patterns associated with an eosinophilic extracellular matrix and reduplicated basement membrane materials, often associated with gene fusions involving the *MYB, MYBL1* and *NFIB* genes. The genomic hallmarks of AdCC are t(6;9) or t(8;9) translocations, resulting in *MYB::NFIB* and *MYBL1::NFIB* fusions, respectively [5, 22] (Fig. 3A-D)**.** The former alteration is found in > 80% of cases and the latter in approximately 5% of cases [5]**.** *MYB/MYBL1* activation due to gene fusion or other mechanisms is a key event in pathogenesis of AdCC [22]**.** Losses of 1p, 6q, and 15q are associated with high-grade tumours, and loss of 14q is seen exclusively in low-grade tumors [23, 24]**.** Next-generation sequencing identified mutations with mostly low level of recurrence in genes of the *FGF/IGF/PI3K*, chromatin remodelling, and *NOTCH* signaling pathways [25, 26]**.** Use of FISH techniques for MYB, MYBL1 or NFIB-rearrangments can be very useful for specific identification of AdCC. Immunohistochemical antibodies to altered MYB can also be used.

Traditional therapeutic agents for patients with advanced, recurrent, or metastatic AdCC have demonstrated poor efficacy in prolonging survival. The presence of *NOTCH* alterations in AdCC associate with poor survival. Patients with these aberrations might potentially benefit from anti-NOTCH drugs, such as bronticuzumab [27].

**Salivary duct carcinoma** (SDC) is an aggressive malignancy resembling mammary ductal carcinoma, most typically with an apocrine phenotype. The most frequent genetic alterations are mutations in *TP53* (55%), *HRAS* (23%), *PIK3CA* (23%), and an amplification of *HER2/neu* (35%) [28]. Due to low recurrency of these mutations, molecular testing for them is not indicated for diagnostic purposes. In diagnostic practice, immunohistochemical androgen receptor positivity is still a useful marker for SDC. *PLAG1* or *HMGA2* rearrangements due to a pre-existing pleomorphic adenoma (PA) can be identified in about half of SDCs [29, 30]. A small subset of SDCs harbors a rearrangement of *ALK* [31]. Given the therapeutic relevance of *ALK* fusions, inclusion of ALK immunohistochemistry in any atypical-looking or androgen receptor poor SDC and high-grade adenocarcinoma, not otherwise specified is recommended. Because systemic therapies targeting androgen receptor, *HER2/neu* amplification, phosphatidylinositol 3 kinase (PI3K) pathway, including mutations of *PIK3CA* and *BRAF* p*.*V600E, and loss of phosphatase and tensin homolog (PTEN) are presently under study, molecular testing for them may become warranted [32–34].

## Molecular alterations with differential diagnostic significance

**Fig. 4:**
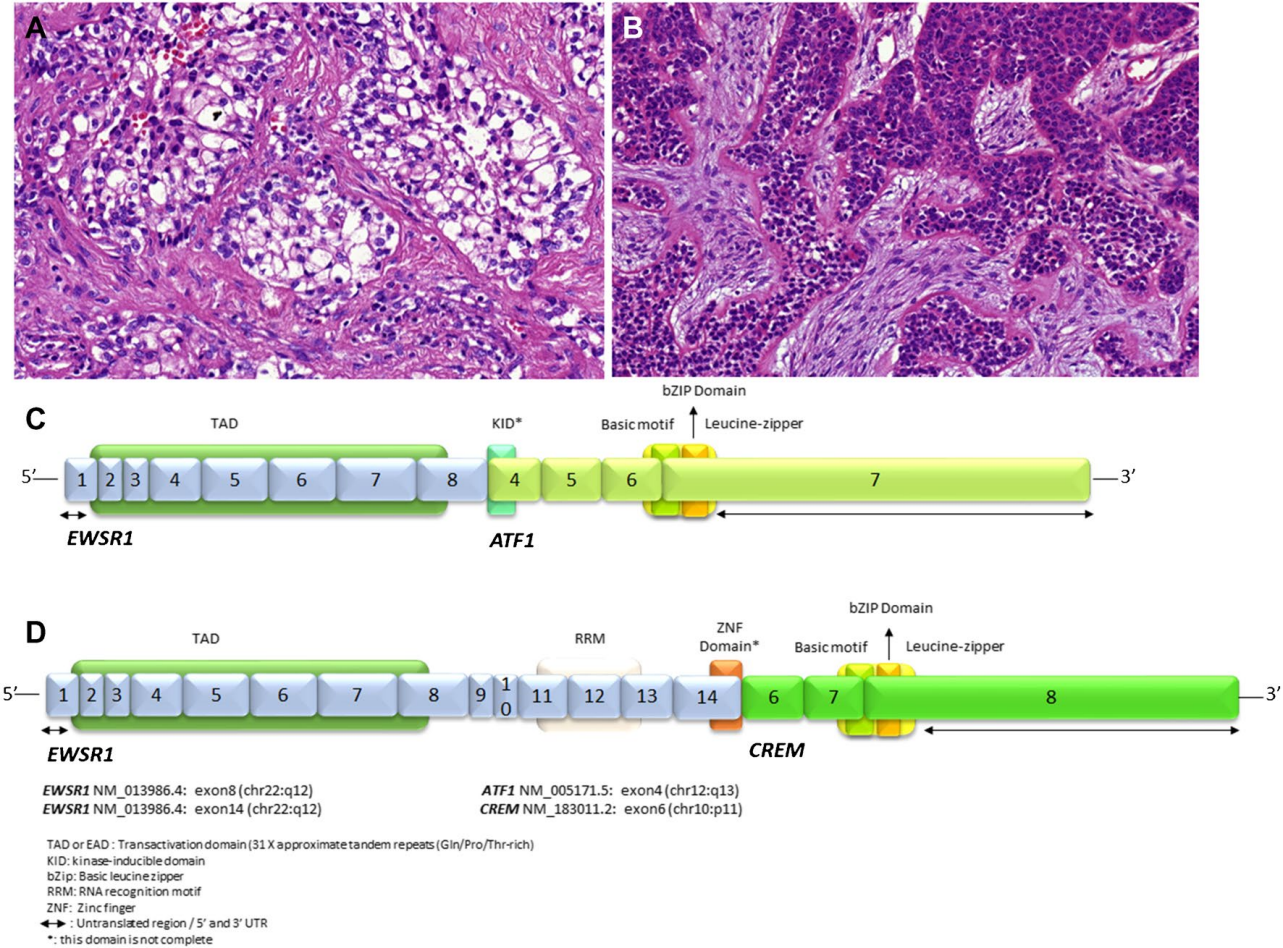
Hyalinizing clear cell carcinoma is characterized by clear cell morphology **(A)** or eosinophilic cells (B) creating solid nestes embedded within two types of stroma hylinized basement membrane-like **(A)** and desmoplastic or fibrocellular **(B)**. The fusions joining of *EWSR1* gene exon 8 with *ATF1* gene exon 4 **(C)** and *EWSR1* gene exon 14 with *CREM* gene exon 6 **(D)** are illustrated. Protein domains are depicted

**Hyalinizing clear cell carcinoma** (HCCC) is a carcinoma composed of clear and eosinophilic cells in a variably hyalinized stroma, usually associated with *EWSR1* rearrangement. Most HCCCs harbor *EWSR1*::*ATF1* gene fusion [8], while *EWSR1::CREM* fusion is less common [35] (Fig. 4A-D). Although the finding of infiltrative nests of low-grade clear tumor cells within a hyalinized or cellular fibrous stroma is quite characteristic of HCCC, some cases may have also abundant mucocytes and/or clear cell differentiation, or the hyalinization is minimal or absent. Differential diagnosis in such cases includes a wide spectrum of salivary gland tumors, namely clear cell mucoepidermoid carcinoma, epithelial-myoepithelial carcinoma, myoepithelial carcinoma, oncocytoma, myoepithelioma, and even metastatic kidney clear cell carcinoma, and the distinction of these benefits from FISH studies [36] on *EWSR1-*rearrangment**.** Conventional immunohistochemistry may also be helpful, but not always conclusive.

**Epithelial-myoepithelial carcinoma** (EMC) is a salivary gland malignancy characterized by biphasic tubular structures and usually a multinodular growth pattern. The histological hallmark of EMC is a biphasic arrangement of inner (luminal) eosinophilic ductal epithelial cells and outer (abluminal) cells which are often clear myoepithelial cells. There are, however, several histologic variants with cribriform, basaloid, sebaceous, apocrine/oncocytic, or double-clear appearances, or with squamous differentiation resulting in differential diagnostic challenges. The salivary gland tumor entities to be differentiated include those with biphasic or/and clear cell morphology, such as adenoid cystic carcinoma, basal cell adenocarcinoma, pleomorphic adenoma, myoepithelial carcinoma, and clear cell carcinoma [37]. Because clear myoepithelial cells can be seen in many benign and malignant salivary gland tumors, the differential diagnosis of epithelial-myoepithelial carcinoma is challenging. Testing for *HRAS* mutations is useful when discriminating EMC from its mimics [38]. Immunohistochemistry is often not conclusive.

**Polymorphous adenocarcinoma** (PAC) (previously known as polymorphous low-grade adenocarcinoma) is a malignant epithelial tumor characterized by cytological uniformity, morphological diversity, and an infiltrative growth pattern. It is predominantly seen in minor salivary glands. Cribriform adenocarcinoma (CAC) was initially reported at the base of the tongue [39] and later in other minor salivary gland locations [40]. CAC is characterized by a multinodular growth pattern separated by fibrous septa, a relatively uniform solid, cribriform and microcystic architecture, and optically clear nuclei. Glomeruloid and papillary structures, peripheral palisading and clefting may be observed. Although CAC and classic PAC have molecular alterations affecting the same *PRKD* gene family, there are notable differences. PACs harbor recurrent *PRKD1* E710D hotspot mutations in > 70% of cases, whereas 80% of CACs display *PRKD1/2/3* fusions with partner genes including *ARID1A*, *DDX3X* or *STRN3* [41, 42]. Histologically, fusion-positive tumors show papillary growth in a high percentage of cases, while arrangement in single cell filing is seen in a low percentage. CACs display a propensity for base of tongue location and frequent (50%) lymph node metastasis compared with the hot spot mutation-related tumors which have a low risk for nodal metastasis. Compared with classic PAC, CAC has a high propensity for location in the base of the tongue and a higher risk for lymph node metastasis [43]. Genetic analysis of *PRKD* genes appears to be useful in characterizing this spectrum of tumors, and identifying those tumors with a high risk for nodal metastasis. Immunohistochemistry may not be conclusive with this regard.

**Pleomorphic adenoma **(PA) is a benign tumor characterized by cytomorphological and architectural diversity with an admixture of ductal and myoepithelial cells usually embedded in a chondromyxoid or fibrous stroma. PAs harbour recurrent translocations or intrachromosomal rearrangements resulting in gene fusions involving *PLAG1* on 8q12 (> 50%) or *HMGA2* on 12q14.3 (10–15%) [44, 45]**.** Antibodies to PLAG1 [46] and HMGA2 [47], respectively, are emerging as practical, sensitive and specific immunohistochemical markers for PA.

Diagnosis of PA is straightforward in most cases but sometimes PA is difficult to differentiate from low-grade epithelial-myoepithelial carcinoma. Molecular testing for *PLAG1/HMGA2* rearrangement versus *HRAS* mutation can be very useful.

**Fig. 5:**
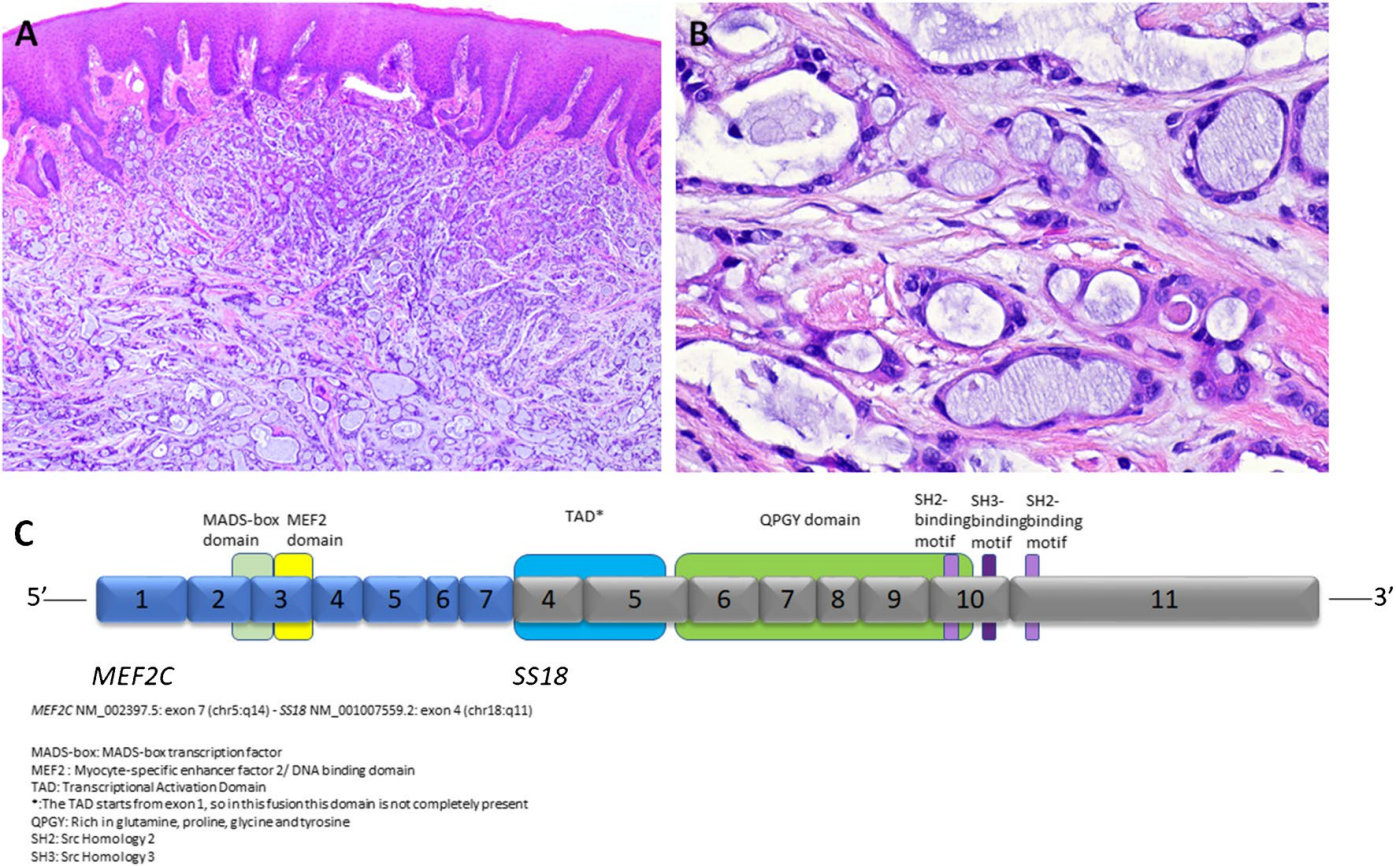
Microsecretory carcinoma is composed of small tubules and microcysts lined by flat intercalated duct-like cells and containing abundant basophilic luminal secretions **(A).** The nuclei are uniform, oval, and lack prominent nucleoli **(B).** The fusion joining of *MEF2C* gene with *SS18* gene is illustrated **(C)**

**Microsecretory adenocarcinoma** (MSA) is a newly identifed low-grade salivary adenocarcinoma (Fig. 5A-C) characterized by distinctive morphology and a specific *MEF2C::SS18* fusion [48].

## Sinonasal neoplasms

Sinonasal tract comprising the nasal cavity, paranasal sinuses and the skull base is a region characterized by a broad spectrum of tumors that exhibit a significant diversity of molecularly defined entities **(**Table 2**).** The recent WHO Classification of Head and Neck Tumors includes new entities in which molecular genetics has an important diagnostic role, including HPV-related multiphenotypic sinonasal carcinoma and SWI/SNF-deficient sinonasal carcinoma and adenocarcinoma [1], and a subset of emerging and developing entities including IDH-mutated malignancies in the category of sinonasal undifferentiated carcinoma. Molecular genetics also plays a diagnostic role in the context of hereditary syndromes that can be manifested in the sinonasal tract.

**Table 2:** Sinonasal tumors reviewed in this article: Immunohistochemical features and molecular genetic data

Tumor type	Immunohistochemistry	Molecular genetics
*Respiratory epithelial lesions – carcinomas*		
Non-keratinizing squamous cell carcinoma	CK, p63/p40, CK5/6, p16	HPV association (20–62%),
		Type 16
		EBV association – rare
		subset *DEK::AFF2*
NUT carcinoma	NUT, p63, CD34	*NUTM1::BRD4/BRD3/NSD3/ (ZNF532, ZNF592)*
SWI/SNF complex-deficient sinonasal carcinoma		
SMARCB1 deficient carcinoma	CK5, p63, CK7, SMARCB1-loss	*SMARCB1* mutation ± *SMARCA2* mutation
SMARCB1 deficient adenocarcinoma	CK7, p40, glypican 3, SALL4, HepPar-1, CDX2, CK20, PLAP and AFP; SMARCB1-loss	*SMARCB1* mutation
SMARCA4 deficient carcinoma	CK, CK7, synaptophysin. Chromogranin, CD56; SMARCA4 loss	*SMARCA4* mutation ± *SMARCA2* mutation
Sinonasal lymphoepithelial carcinoma	EBV	EBV
Sinonasal undifferentiated carcinoma (SNUC)	diagnosis per exclusionem, a subset IDH1/2	*IDH wild type and IDH1/2* mutations
HPV-associated multiphenotypic sinonasal carcinoma	Biphasic, S100, SOX10, p16	HPV – mainly serovariety 33

Excluded entities without diagnostic molecular genetic characteristics

Adapted from:

WHO Classification of Tumours Editorial Board. Head and neck tumours. Lyon (France): International Agency for Research on Cancer; forthcoming. (WHO classification of tumours series, 5th ed.; vol. 9). https://publications.iarc.fr

**Non-keratinizing squamous cell carcinom**a (NKSCC) with *DEK::AFF2* gene fusion. Sinonasal NKSCC is in 36–58% driven by transcriptionally active HPV, most commonly type 16 [1]. *DEK::AFF2* carcinoma is currently classified as an emerging entity under NKSCC category, localized especially in the sinonasal tract but also few cases were reported in the middle ear [49, 50]. Despite bland looking morphology this tumor behaves in and aggressive fashion with high-risk of local recurrence, nodal metastatic dissemination and distant spread. FISH testing for *DEK*- or *AFF2* rearrangements is essential for diagnosis. *DEK::AFF2* carcinoma has an excellent response to immune checkpoint inhibitors (ICI) – anti-PD-L1 [49].

**NUT Carcinoma** is a highly aggressive, mostly lethal malignancy with a monotonous poorly differentiated morphology.

NUT carcinoma is genetically characterized by rearrangement of *NUTM1* gene on 15q14 [51]. The NUT gene is physiologically expressed in mature spermatogonia. The most common fusion partners of NUT are genes involved in transcription and chromosome regulation belonging to the BET family (*BRD2, BRD3, BRD4* and *BRDT)*[52]. In 75% of cases the fusion partner of *NUTM1* is the *BRD4* gene (in 19p13.1), and in 15% the *BRD3* gene (in 9q34.2) [53, 54]. FISH techniques or other molecular testing may be used to demonstrate NUTM1- and the various BRD-rearrangements. However, the monoclonal NUT antibody is highly specific for NUT carcinoma [55]**,** and offers a simple and reliable way to diagnose this malignancy.

In a subset of cases, *NUT* is fused with non-BRD genes. In 6% of cases fusion involves the *NSD3* gene (in 8p11.23) [56], while in 2% of cases, genes for zinc finger containing proteins, (*ZNF532* on 18q21.32*,* or *ZNF592* on 15q25.3) are involved in *NUT* fusion [57].

There is no known effective treatment for NUT carcinoma which has a median survival of 6.7 months [58]. The first targeted drugs for NUT carcinoma were histone deacetylase inhibitors (HDACi) (vorinostat) and BET inhibitors (BETi) [59].

## SWI/SNF complex deficient sinonasal carcinoma and other malignancies

The chromatin remodeling Switch/Sucrose non-fermentable complex (SWI/SNF) is a pleomorphic complex of over 20 tumor suppressors [60]. There are five different subtypes of SWI/SNF complex deficient sinonasal/base of skull malignancies, including SMARCB1-deficient sinonasal carcinoma; SMARCB1-deficient sinonasal adenocarcinoma; SMARCA4-deficient sinonasal carcinoma, a subset of SMARCA4-deficient teratocarcinosarcomas and poorly differentiated chordomas.

These tumors are usually poorly differentiated or undifferentiated malignancies with a highly aggressive clinical course and poor outcome.

**Fig. 6:**
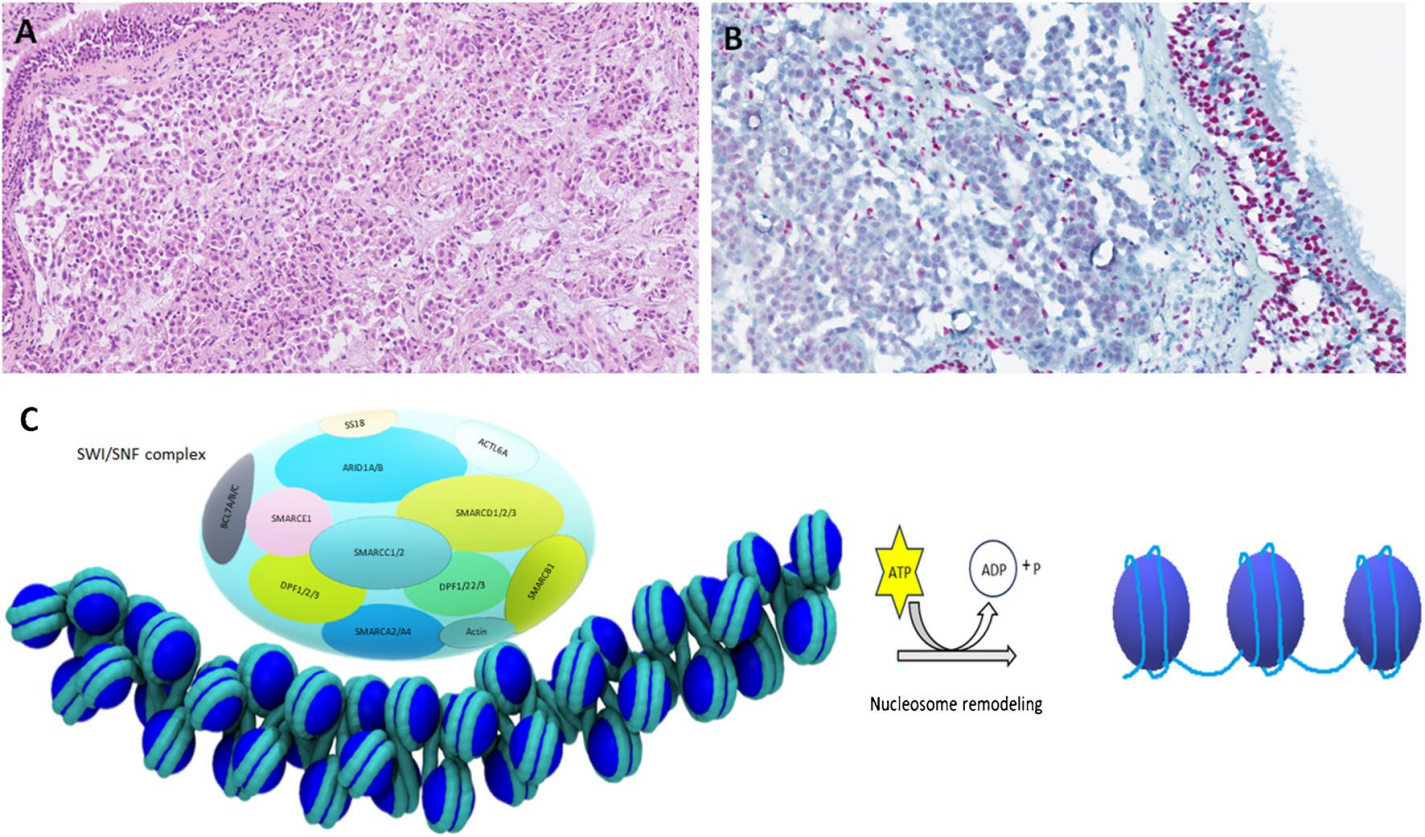
SMARCB1 deficient adenocarcinoma of nasal cavity composed of eosinophilic cells **(A)** devoid of INI1 immunostaining **(B).** SWI/SNF complex is depicted **(C)**

The mortality of SMARCA4-deficient sinonasal carcinomas is higher than in other tumors of this family [61, 62]. SWI/SNF mutations are involved in many cancer-related pathways mostly related to epigenetic alterations (Fig. 6A-C). SWI/SNF complex deficient malignancies can be preferentially identified by immunohistochemical antibodies to SMARCB1 (INI1) and SMARCA4 (BRG1) which are sensitive diagnostic tools [63].The therapeutic option rests in local control of the disease with polychemotherapy and radiotherapy [61, 62].

Recent findings indicate a promising role for immunomodulators and immune checkpoint inhibitors as potential drugs in patients with SWI/SNF related malignancies [60, 64]. Tumor cells with loss of *SMARCB1* demonstrate a constitutive EZH2 activation, and EZH2 inhibitors may modulate tumor immunogenicity and anti-tumor immune response [60, 64, 65].

**Sinonasal undifferentiated carcinoma** (SNUC) is a high-grade epithelial neoplasm without signs of cell differentiation, and the diagnosis is made only by exclusion of other sinonasal and non-sinonasal malignancies. Many undifferentiated epithelial neoplasms in previous WHO classifications were included under this term until advances in molecular pathology allowed for their identification as proper entities. Recently, isocitrate dehydrogenase 1 or 2 (*IDH1/2*) mutations were identified in a subset of SNUC [66, 67] including three main hotspot mutations of *IDH1 R132, IDH2 R140* and *IDH2 R172* [68]. Monoclonal or multi-specific antibodies for immunohistochemical detection of IDH1/2 mutations represent a cheaper alternative to molecular genetic testing for these mutations. However, immunohistochemistry lacks the ability to detect the full spectrum of IDH1/2 mutations [69].

SNUCs are aggressive diseases with poor outcome. The standard therapeutical approach is a combination of surgery, chemotherapy and radiation [70]. Tumors with *IDH2*-mutations show better outcome than other SNUCs [70]. *IDH* mutations provide alternative therapeutic options including selective small molecule inhibitors (e.g. Enasidenib for *IDH2* mutations and Ivosidenib for *IDH1* mutations).

**HPV-associated multiphenotypic sinonasal carcinoma** (HMSC) is an epithelial tumor almost exclusively localized in sinonasal tract and harboring high-risk HPV [71]. The most common serovariety is type 33. P16 immunohistochemistry can be used to identify the HPV-association. HMSC is histologically very pleomorphic and may mimic various salivary and non-salivary tumor types. The histological appearance of HMSC is usually high-grade and associated with destructive growth and propensity for local recurrence. Despite the aggressive appearance, HMSC has low metastatic potential and little tendency to lethal behavior [72].

## Soft tissue neoplasms with head and neck predilection

### Molecular alterations with differential diagnostic significance

The head and neck can be host to a wide range of soft tissue neoplasms with molecular findings, but a discussion of all these entities is beyond the scope of this review. We have included a selection of soft tissue tumors, often of recent molecular definition, that harbor diagnostically obviously useful molecular alterations. All discussed entities are listed in Table 3.

**Table 3:** Soft tissue tumors of Head and Neck reviewed in this article: Location, immunohistochemical features and molecular genetic data

Tumor type	Typical location(s)	Immunohistochemistry	Molecular genetics
*Fibroblastic and myofibroblastic tumors*			
Inflammatory myofibroblastic tumor	larynx, sinonasal area, oral cavity	ALK, ROS1, SMA	*ALK/ROS1-*rearrangements
*Skeletal muscle tumors*			
Rhabdomyosarcomas (RMS)			
* Alveolar RMS*	oral cavity, sinonasal area	PAX3/7-FOXO1, MYOD1, desmin, myogenin	*PAX3::FOXO1 (*or *PAX7::FOXO1)*
* VGLL2/3, NCOA1, CITED1 rearranged RMS*	rarely	MYOD1, desmin, myogenin	*VGLL2/3, SRF, TEAD1, NCOA2, and CITED2* rearrangements
* MyoD1 mutant RMS*	rarely	MYOD1, desmin, myogenin	*MYOD1 p.Leu122Arg* gene mutation
* TFCP2 rearranged RMS*	mandible, maxilla	MYOD1, desmin, myogenin, CK, ALK (50%)	*EWSR1/FUS::TFCP2, ALK* alterations
*Tumors of uncertain differentiation*			
*GLI1*-altered soft tissue tumor	tongue, submandibular gland, soft tissues of the neck	**GLI1**, variable S100, MDM2, CDK4, STAT6 SMA and CK	*GLI1* rearrangement or *GLI1* amplification
*Undifferentiated small round cell sarcomas of bone and soft tissue*			
* Adamantinoma like ES*	salivary glands, thyroid gland, and sinonasal area	CK, p40, p63, CD99, NKX2.2	*EWSR1::FLI1*
*Others*			
Biphenotypic sinonasal sarcoma	Sinonasal	SMA, S100, variably desmin, MyoD1	*PAX3 gene rearrangements (NCOA1, NCOA2, FOXO1, FOXO6, WWTR1* and others)
Ectomesenchymal chondromyxoid tumor	Tongue	GFAP, variably S100, desmin, SMA, CK, EMA	*RREB1::MRTFB* (formerly called *MKL2)* fusions
* NTRK*-rearranged spindle cell neoplasms	Broad distribution including craniofacial bones	Variably S100, CD34, CD30	*NTRK1/2/3, BRAF, RAF1, RET, MET* and other kinase fusions, rarely *BRAF* or *EGFR* mutations
* EWSR1/FUS::POU2AF3* sarcomas	Sinonasal, rarely craniofacial bones	Variably CK, GFAP, S100 protein, neuroendocrine markers, SATB2	*EWSR1/FUS::POU2AF3* (formerly called *COLCA2)* fusions

Excluded entities without diagnostic molecular genetic characteristics and entities not occurring predominantly in the head and neck

Adapted from:

WHO Classification of Tumours Editorial Board. Head and neck tumours. Lyon (France): International Agency for Research on Cancer; forthcoming. (WHO classification of tumours series, 5th ed.; vol. 9). https://publications.iarc.fr

Jo VY, Demicco EG. Update from the 5th Edition of the World Health Organization Classification of Head and Neck Tumors: Soft Tissue Tumors. *Head Neck Pathol.* 2022;16:87–100

WHO Classification of Tumours Editorial Board. Soft tissue and bone tumours. Lyon (France): International Agency for Research on Cancer; 2020. (WHO classification of tumours series, 5th ed.; vol. 3). https://publications.iarc.fr/588

**Biphenotypic sinonasal sarcoma** (BSS) is a low-grade mesenchymal neoplasm with neurogenic and myogenic differentiation localized exclusively in the sinonasal region. BSS is molecularly defined by rearrangement of *PAX3* gene [73]. The most common fusion is *PAX3::MAML3* in more than half of the cases, with alternative fusion partners including *NCOA1, NCOA2*, *FOXO1*, *FOXO6* and *WWTR1* [74–76].

BSS are usually low-grade tumors with frequent local recurrence, sometimes many years after diagnosis [77]. Three cases of BSS with high-grade transformation have been published [78–80], one of which developed into rhabdomyosarcoma (RMS). Molecular testing of BSS and RMS showed similar gene fusions including *PAX3::FOXO1* and *PAX3::NCOA1* [74].

**Nasal chondromesenchymal hamartoma** (NCMH) is a pediatric benign mesenchymal sinonasal tumor which in a subset of cases is associated with *DICER1* syndrome [81]. NCMH is usually an indolent tumor but when syndromic, patients have an increased risk for pleuropulmonary blastoma, ovarian sex cord-stromal tumor, renal tumors, genitourinary embryonal rhabdomyosarcoma, brain tumors and ciliary body medulloepithelioma. When diagnosing NCMH, genetic testing for germline *DICER1* is recommended.

**Inflammatory myofibroblastic tumor** (IMT) is a low-grade neoplasm which in approximately 15% of cases arises in the head and neck with favorable prognosis and very infrequent recurrences [82, 83]. IMT is driven by constitutive activity of ALK or other receptor tyrosine kinase. Immunostaining for ALK-1 serves as a valuable screening tool, and *ALK* rearrangement can be confirmed with FISH techniques. Rare cases of IMT with fusions of other tyrosine kinase genes suggest that in cases of ALK-1 immunonegativity targeted RNA-sequencing would be a more suitable test.

**Ectomesenchymal chondromyxoid tumor** (ECT) (aka *RREB1::MRTFB-*rearranged neoplasm) is a tumor of uncertain malignant potential located predominantly in the tongue and rarely arising in extraglossal locations [84, 85]. The immunoprofile is non-specific, and therefore molecular testing is the preferred method when diagnosing ECT. In most cases it is characterized by a *RREB1::MRTFB* fusion, while *EWSR1* gene rearrangement is seen in a small subset of cases [86, 87]. These tumors are genetically and histologically linked to soft tissue myoepithelial tumors [86] and may be mistaken for other mesenchymal tumors with dominant spindle cell morphology [88]. ECT usually follows benign course with no metastasis. Surgery is curative in most cases, even though local recurrences may occur [84, 85].

**GLI1-altered soft tissue neoplasms** are tumors of uncertain histogenesis, epithelioid morphology and non-specific immunoprofile, presenting in the head and neck in 40% of cases. In two thirds of cases these tumors harbor *GLI1* fusions including *ACTB::GLI1*, *PTCH1::GLI1, MALAT1::GLI1*, and *DERA::GLI1*, while the rest harbor *GLI1* amplification [1, 89, 90]. The various fusion partners to GLI1 are best determined by targeted RNA-sequencing but GLI1 amplification can also be detected by FISH. Awareness of a potential co-amplification of neighboring genes (*CDK4, MDM2, DDIT3, *etc*.)* on chromosome 12 detectable by FISH has to be taken into account [91]**.** GLI1 immunostaining shows high specificity and good sensitivity for GLI1-rearranged mesenchymal tumors. However, the sensitivity is unsatisfactory in plexiform fibromyxoma [92]. Biologic behavior varies from indolent tumors to metastasizing malignancies. Local recurrence or distant spread may appear in approximately 20% of cases [90, 93]. Potential targeted therapeutic options in *GLI1*-altered neoplasms are sonic hedgehod (Shh) signaling pathway inhibitors [94].

**Rhabdomyosarcomas **(RMS) are a clinically, prognostically and biologically heterogeneous group of tumors categorized morphologically into four main subtypes [95]. However, molecular-genetic findings delineate six distinct subtypes; embryonal RMS with unknown driver mutations/fusions, alveolar RMS with *FOXO1* fusions, *MYOD1-*mutated RMS with *MYOD1* activating mutations, *VGLL2/VGLL3/NCOA2*-rearranged RMS, pleomorphic RMS with a complex genetic background, and finally *TFCP2*-rearranged RMS with *EWSR1* or *FUS* fusion partners [96]. Immunohistochemically, spindle cell/sclerosing RMS are usually positive (at least focally) for cytokeratins and myogenic markers, mainly MyoD1, myogenin, desmin and PAX7.

***VGLL2/VGLL3/NCOA2*-rearranged **RMS belong to spindle cell/sclerosing RMS, primarily affecting newborns or infants with head and neck predilection. The characteristic molecular genetic events involve *VGLL2::CITED3, VGLL2::NCOA2, TEAD1::NCOA2,* or *SRF::NCOA2* gene fusions [97, 98]. Recently, novel *VGLL3* rearrangements with *TCF12, EP300* and *PPARGC1A* as fusion partners have been described [99]. Complete surgical resection is usually curative, while RMS-type chemotherapy is recommended for unresectable cases.

***TFCP2*-rearranged RMS** (*TFCP2*-RMS) are spindle cell/sclerosing and very aggressive mesenchymal tumors with rhabdomyoblastic differentiation and *EWSR1/FUS::TFCP2* rearrangements [100]. In a subset of cases, hemizygous deletion or amplification of the *ALK* gene are described [101]. *TFCP2*-RMS tumors are predominantly localized in the jaws and the skull of young adults. The tumors have a rapid clinical course with a poor prognosis of 3-year overall survival at 28% [102]. Treatment options include surgery. chemotherapy and radiotherapy. The combination of radiation and *ALK* inhibitors has shown partial response in anecdotal cases [103, 104].

**Adamantinoma-like Ewing sarcoma** (ALES) is a controversial variant of Ewing sarcoma (ES) defined by the presence of t(11;22) and *EWSR1::FLI1* fusion [105]. It is speculated whether they represent an epithelial or mesenchymal neoplasm, as they commonly express epithelial markers (pancytokeratins and p63/p40) as well as ES-related markers (CD99 and NKX2.2) [106]. Many tumors are treated with surgery and adjuvant polychemotherapy according to ES-specific protocols.

***NTRK*- and other kinase gene-rearranged spindle cell neoplasms** encompass a wide morphological spectrum ranging from benign-appearing to high-grade tumors, consisting of spindled cells often immunopositive for S100 protein and CD34, and resembling infantile or adult-type fibrosarcoma, malignant peripheral nerve sheath tumor, or lipofibromatosis-like neural tumor. Superficial and deep soft tissues including salivary glands, as well as the skeletal component of the head and neck, are some of the more common locations of these tumors [107, 108].

While *ETV6::NTRK3* is a well-established genetic driver of infantile fibrosarcoma, other fusions of *NTRK1/3* and various other kinase genes including *MET*, *RET*, *RAF1*, or *BRAF* have been implicated in the pathogenesis of these tumors. Most cases are only locally aggressive and distant metastasis is an uncommon event in high-grade cases [108]**.** PanTrk immunohistochemistry seems to be a useful screening tool in *NTRK1/3* rearranged cases with an overall sensitivity of 88% [109]. FISH detection of *ETV6::NTRK3* is sufficient to confirm the diagnosis of infantile fibrosarcoma. However, given the number of possible kinase genes and their fusion partners when other tumor entities come into question, targeted RNA-sequencing is the method of choice [108]. Importantly, small inhibitory molecules targeting NTRK and RET have been used in the treatment of locally advanced inoperable tumors or cases with metastatic spread.

***EWSR1/FUS::POU2AF3(COLCA2)*** **sarcomas** are newly recognized aggressive neoplasms with tendency to both local recurrence and metastatic spread despite multimodal treatment. They affect adult patients and commonly arise in the head and neck, particularly within the sinonasal tract. Their morphological spectrum of these tumors is wide from spindle cells, through biphasic tumors with spindled and round cells with features of neuroendocrine differentiation, to purely round cell tumors with high nuclear grade [110–112]. Approximately half of the cases display pan-cytokeratin positivity [110–112], possibly leading to diagnostic confusion with synovial sarcoma. As *EWSR1* fusions with various partners are frequent in soft tissue tumors, FISH analysis of an *EWSR1* break is not a sufficient diagnostic test. Instead, targeted RNA-sequencing is advised to render the correct diagnosis.

## HPV- And EBV-Associated tumors of the nasopharynx and the oropharynx

The expression of late viral genes plays a role in the pathogenesis of epithelial and lymphoproliferative neoplasms of the oropharyngeal and nasopharyngeal areas. Only a considerably small subset of patients who acquired the infection during their lifetime develop any related tumor, highlighting the major influence of other genetic and environmental factors in their pathogenesis.

Nasopharyngeal carcinoma (NPC) is a rare neoplasm with high incidence in high-risk (endemic) populations including ethnic Chinese (southern China and Singapore) and Inuits of Alaska, Canada and Greenland. Low-risk (non-endemic) populations with rare occurrence of NPC include Europeans and Americans of Caucasian ethnicity [1]. In most cases of non-keratinizing NPC in high-risk populations, EBV infection is an early pathogenetic event [113]. Other important pathogenetic influences include genetic and environmental factors [114]. In low-risk populations, fewer cases are EBV positive, while high-risk HPV positive and virus-negative cases, particularly in keratinizing NPC are seen [115, 116]. In-situ hybridization with EBV-encoded small RNAs (EBER) or RNA-ISH for high-risk HPV can be used to detect the viral load in tumor cells. Immunohistochemistry for LMP-1 has low sensitivity and is not recommended. Serum EBV DNA and antibody levels have prognostic value, and patients with high viral load fare worse than those with lower load or absence of viral DNA [114]. In EBV- and HPV-positive cases patients have shown better outcome than in virus-negative cases [115].

HPV-associated squamous cell carcinoma of the oropharynx has an increasing incidence especially in developed countries. In North America and Northern Europe it is more prevalent now than the HPV-independent oropharyngeal squamous cell carcinoma [117]. High-risk HPV16 is responsible for most cases of HPV-associated carcinoma [118]. The patients are generally younger, have a higher socioeconomic status and have a better prognosis (5-year overall survival of 80%) than patients with HPV-independent carcinoma [1, 119, 120]. However, similar to HPV-independent cases, patients with HPV-associated squamous cell carcinoma are often active or past smokers [119]. The HPV-associated carcinomas most commonly originate from the squamous epithelium of tonsillar crypts and lack keratinization [121]. Histologic grading is not recommended as it does not carry significant prognostic value. In addition, the HPV-associated neuroendocrine carcinoma is a distinct aggressive tumor type that must be differentiated from squamous cell carcinoma. This can be facilitated by positive immunohistochemical staining for neuroendocrine markers such as synaptophysin, chromogranin and INSM1. Immunohistochemistry for the surrogate marker p16 is sufficient to detect HPV infection in most cases [122]. Strong diffuse positivity in tumor cell cytoplasm and nuclei must be present in more than 70% of cells to diagnose the lesion as positive. In problematic instances (equivocal p16 positivity, discrepancy between morphology and immunoprofile), PCR genotyping or in-situ hybridization methods can be employed to confirm the presence of high-risk HPV in the tumor.

Extranodal NK/T-cell lymphoma, nasal type is an aggressive tumor that infiltrates and destroys the nasal cavity, the nasopharynx, the oropharynx and structures of the oral cavity. It belongs to EBV-positive lymphoproliferations, and in-situ hybridization for EBER is positive in most viable tumor cells [1]. While a 5-year overall survival can be achieved using chemoradiotherapy in a majority of patients, the detection of persistent post-treatment serum EBV DNA suggests a worse prognosis [123].

EBV-associated smooth muscle tumor and EBV-positive mucocutaneous ulcer are two indolent EBV-positive lesions that can be found in the oral or nasal cavities, pharynx and larynx of immunosuppressed patients. Both display positivity for EBER in-situ hybridization and can be treated with conservative surgical removal. In addition, some cases might regress spontaneously with decreased immunosuppression [1, 124, 125].

In conclusion, a tremendous development has taken place in the classification of head and neck tumors with widespread application of molecular testing. In many cases, an expanding spectrum of immunohistochemical surrogates helps with histopathological diagnosis, and also with rapid identification of diagnostic molecular abnormalities. Therefore, pathologists currently benefit from the molecular underpinnings of tumors while still making diagnoses largely based on histology and immunohistochemistry.

## Data Availability

Data supporting the findings of this study are available within the article. The complete datasets generated during and/or analyzed during the current study are available from the corresponding author upon reasonable request.
